# Artificial intelligence improves radiologist workflow and assessment of image quality accuracy

**DOI:** 10.6026/973206300221322

**Published:** 2026-03-31

**Authors:** Rishabh Yadav, Mukesh Kumar, Ifath Nazia Ghori, Abhijeet Alok, Amrita Pandita Bhatia, Santosh Kumar Kotnoor, Rahul Tiwari

**Affiliations:** 1 Department of Radiology, Prasad Medical College, Lucknow, Uttar Pradesh, India; 2 Department of Oral & Maxillofacial Surgery, Shree Bankey Bihari Dental College, Masuri, Ghaziabad, Uttar Pradesh, India; 3 Department of Computer Science, Jazan University, Jazan, Saudi Arabia; 4 Department of Oral Medicine and Radiology, Sarjug Dental College and Hospital, Darbhanga, Bihar, India; 5 Department of Prosthodontics (Crown and Bridges and Implantology), YCCM and RDF's Dental College and Hospital, Ahilyanagar, Maharashtra, India; 6 Department of Oral Pathology and Microbiology, HKES S. Nijalingappa Institute of Dental Science and Research, Kalaburgi, Karnataka, India; 7 Department of Oral and Maxillofacial Surgery, Narsinhbhai Patel Dental College and Hospital, Sankalchand Patel University, Visnagar, Gujarat, India

**Keywords:** Artificial intelligence (AI), radiology workflow, image quality assessment (IQA), picture archiving and communication system (PACS) integration, turnaround time

## Abstract

Increasing imaging volumes and workflow pressures make consistent image quality assessment (IQA) challenging for radiologists in routine clinical practice. Artificial intelligence (AI) systems have recently been introduced to support quality control tasks, yet real-world evidence on their impact on radiologist workflow and diagnostic performance remains limited. Therefore, it is of interest to assess the effect of AI-IQA module of PACS/worklist on radiological workflow and accuracy in identifying suboptimal CT/MRI tests. Workflow (IQA time, TTFR and TAT) and IQA performance (sensitivity, specificity, kappa) metrics were evaluated in two groups (before and after 8 weeks of AI integration) and compared to an expert reference standard. AI integration lowered median IQA task time/examination and improved report turnaround time and improved radiologist sensitivity to suboptimal IQA without affecting specificity. Thus, we show AI-assisted IQA as a scalable tool to enhance the quality control and operation performance in the routine clinical practice.

## Background:

Radiology departments experience the problem of the growing volume of imaging and growing complexity of the examination that puts a strain on the workflow, making the repeatability of routine image quality assessment (IQA) a problem. The field of AI has also expanded to operational areas including triage, reporting assistance and quality control and clinical implementation models have focused on the fact that operational value is achievable only by integration and governance and not by isolated model performance [1]. It has been pointed out that standards-based workflow integration is critical to secure implementation and scalable implementation in the common PACS settings [2]. There are previous real-world investigations demonstrating that AI-powered prioritization can lead to a reduction in the time to diagnosis and enhance turnaround schedule of picked urgent discoveries, demonstrating that workflow-focused AI can be converted into quantifiable efficiency advantages when integrated in a suitable manner [3, 4]. Simultaneously, automated methods based on IQA have been introduced to CT and MRI to detect artifacts, noise, motion and poor acquisitions, although little evidence has been found on how they impact radiologist accuracy and normal reporting performance [5]. Therefore, it is of interest to assess the role of artificial intelligence on imaging diagnosis and workflow on radiologists.

## Materials and Methods:

A quasi experimental, before and after study on regular adult CT and MRI studies, as interpreted in clinical practice, was carried out in a single center. The pre-AI phase (8 weeks) was characterized by regular IQA the work of radiologists and the post-AI phase (8 weeks) was characterized by an AI-IQA module built into the PACS/worklist. The system automatically created an examination-level quality label (optimal vs suboptimal), a quality driver dominant (motion/noise/coverage/positioning) and a confidence score shown as an IQA banner at case opening and as a worklist filter flagged studies. Radiologists were still in full control to accept or override AI outputs. Radiologist IQA task time per examination (micro-log plus audit markers), time-to-first-read (TTFR) and report turnaround time (TAT) were regarded as the workflow endpoints. The appropriate endpoints of IQA were radiologist sensitivity and radiologist specificity when diagnosing poor examinations and consensus with an expert reference standard, based on consent of two senior radiologists regarding technologist QA notes. Examinations were not included when pediatric, interventional, or not finished because of non-technical interruption, or the lack of the timestamp data. The statistical comparisons were made on the basis of χ^2^-tests when categorical variables were concerned and on the basis of Mann and Whitney U-tests when time results were non-normally distributed and were measured multi-variable regression were used to adjust the analysis in terms of modality, type of shift and volume on a daily basis.

## Results:

There were an equal number of examinations of 4,800 analyzed in the pre-AI and the post-AI period. There were no significant differences in patient age, sex distribution, modality mix (average two-thirds CT and one-third MRI) and clinical setting across periods, which suggests the presence of minimal case-mix imbalance. The percentage of reference-standard suboptimal image quality also did not change significantly (around 10 percent) trying to justify comparability of quality burden at baseline. Those similarities minimize the possibility that the workflow and accuracy changes are motivated by variations in the composition of examinations Table 1 (see PDF). Introduction of AI minimized the median time spent on IQA tasks per study by radiologists, in addition to increasing TTFR and report turnaround time. Notably, the radiologist sensitivity to detect suboptimal examinations improved significantly, but specificity remained constant, which implies better detection and fewer false positives. Inter-rater agreement with the reference standard changed to substantial to almost excellent levels. The secondary outcomes demonstrated moderate but statistically significant improvements in repeat imaging and quality-related addenda so that their downstream clinical benefits are likely to be clinically meaningful Table 2 (see PDF).

## Discussion:

This paper shows that the consistent implementation of an AI-based image quality assessment tool as a part of a regular PACS/worklist workflow may provide operational and interpretative advantages in everyday radiology. The decreased time of IQA tasks indicates that artificial intelligence support is capable of standardising the preliminary quality control process and minimise the necessity of radiologists manually spending time on searching the movement, noise or insufficient coverage or positioning constraints in two or more series. This is in line with the rest of the evidence base saying that AI may enhance efficiency in medical imaging but effect sizes differ across settings, uses and integration strategies [6, 7, 8, 9-10]. We evaluated increased turnaround time in report creation as an indicator of not only less cost in cognitive switching, but faster recognition of quality restrictions and subsequent interpretations, as well as more consistent documentation. One of the conclusions was that radiologist sensitivity had improved in the detection of inappropriate examinations and specificity was maintained. This has clinical significance since quality constraints may decrease diagnostic certainty, postpone reports and escalate subsequently used resources. Recent CT-oriented studies prove that deep learning would be able to effectively classify the adequacy of acquisitions and determine the key quality drivers, such as the respiratory phase problems, that could not be assessed in a consistent manner in the high-volume scenario [7, 11 12-13].

In a comparable manner, MRI studies have demonstrated that deep learning is reliable in the detection and grading of the artifacts and automated quality grading is a feasible tool that can be efficiently used in a clinical environment [8, 14]. These technical validations are furthered by our results that indicate that in a situation where AI outputs are incorporated into the routine workflow, the performance of radiologists and their agreement with expert reference performance increase. The noted decreases in repeat imaging and quality-related addenda indicate that AI-based IQA can also potentially exert an effect on downstream clinical pathways. Although the current study cannot confirm any causality of these secondary endpoints, the direction is likely to be true. Superior early identification of low quality can be used to clarify at once, other limitation reporting being made standard and even more effective corrective interventions in borderline cases. Moreover, AI-based reconstruction and image enhancement techniques have been reported to enhance perceived image quality and to decrease the time of acquisition which can be used in conjunction with AI-IQA systems in combined pipelines in the future [13]. Introducing AI functionality in structured formats into radiology reports has also been demonstrated to decrease reporting time and enhance report quality, which supports the notion that workflow design is the key to achieving the benefits of clinical AI [11, 12]. However, there are still issues of implementation. The use of workflow AI has to be regulated to avoid automation bias, supervision of radiologists and transparency of model constraints. Interoperability and standards-based integration are also necessary in an operational deployment to ensure that the new interfaces or additional steps are not made to counteract the efficiency benefits [15]. Multicenter research in the future ought to evaluate the generalizability, incorporate human factors (e.g., cognitive load) and determine the cost-effectiveness of a variety of imaging volumes and staffing models.

## Conclusion:

Image quality assessment aided by AI and embedded into the normal PACS/ worklist workflow enhanced the efficiency and timeliness of reporting by radiologists under normal clinical practice. There was no loss of specificity and radiologist accuracy and agreement to identify suboptimal examinations were improved. The value of the clinical usefulness of workflow-integrated AI-IQA systems as a component of scalable radiology quality assurance is supported by these findings.

## Advancement to Knowledge:

This study demonstrates that integration of an artificial intelligence-based image quality assessment system directly into the PACS workflow can significantly improve radiologist efficiency and sensitivity in detecting suboptimal imaging examinations without compromising diagnostic specificity in routine clinical practice.
